# Evaluation of circulating microRNAs as non-invasive biomarkers in the diagnosis of ovarian cancer: a case–control study

**DOI:** 10.1007/s00404-021-06287-1

**Published:** 2021-12-10

**Authors:** Kai Berner, Marc Hirschfeld, Daniela Weiß, Gerta Rücker, Jasmin Asberger, Andrea Ritter, Claudia Nöthling, Markus Jäger, Ingolf Juhasz-Böss, Thalia Erbes

**Affiliations:** 1 Department of Obstetrics and Gynecology, Medical Center, University of Freiburg, Hugstetterstr. 55, 79106 Freiburg, Germany; 2 Faculty of Medicine, University of Freiburg, Freiburg, Germany; 3 Institute of Veterinary Medicine, Georg-August-University Goettingen, Göttingen, Germany; 4 Institute of Medical Biometry and Statistics, Medical Center, University of Freiburg, Freiburg, Germany

**Keywords:** microRNAs, Ovarian cancer, Liquid biopsies, Urine, Disease biomarker, Urinary microRNAs, Hypoxia, Acidosis

## Abstract

**Purpose:**

Ovarian cancer is the seventh most frequent form of malignant diseases in women worldwide and over 150,000 women die from it every year. More than 70 percent of all ovarian cancer patients are diagnosed at a late-stage disease with poor prognosis necessitating the development of sufficient screening biomarkers. MicroRNAs displayed promising potential as early diagnostics in various malignant diseases including ovarian cancer. The presented study aimed at identifying single microRNAs and microRNA combinations detecting ovarian cancer in vitro and in vivo.

**Methods:**

Intracellular, extracellular and urinary microRNA expression levels of twelve microRNAs (let-7a, let-7d, miR-10a, miR-15a, miR-15b, miR-19b, miR-20a, miR-21, miR-100, miR-125b, miR-155, miR-222) were quantified performing quantitative real-time-PCR. Therefore, the three ovarian cancer cell lines SK-OV-3, OAW-42, EFO-27 as well as urine samples of ovarian cancer patients and healthy controls were analyzed.

**Results:**

MiR-15a, miR-20a and miR-222 showed expression level alterations extracellularly, whereas miR-125b did intracellularly across the analyzed cell lines. MicroRNA expression alterations in single cell lines suggest subtype specificity in both compartments. Hypoxia and acidosis showed scarce effects on single miRNA expression levels only. Furthermore, we were able to demonstrate the feasibility to clearly detect the 12 miRNAs in urine samples. In urine, miR-15a was upregulated whereas let-7a was down-regulated in ovarian cancer patients.

**Conclusion:**

Intracellular, extracellular and urinary microRNA expression alterations emphasize their great potential as biomarkers in liquid biopsies. Especially, miR-15a and let-7a qualify for possible circulating biomarkers in liquid biopsies of ovarian cancer patients.

**Supplementary Information:**

The online version contains supplementary material available at 10.1007/s00404-021-06287-1.

## Background

Ovarian cancer (OC) is the seventh most frequent form of malignant diseases in women worldwide with an incidence of nearly 300,000 in 2018 [1]. Due to difficulties in detecting it at an early stage, over 150,000 women die from it every year, placing it as the most lethal malignant gynecologic disease in terms of mortality [2]. Over 70 percent of OC patients are diagnosed at an advanced, incurable stage (stages III and IV) with a five-year survival rate of 29% and only 15% are diagnosed when the disease is still localized [3, 4]. Established diagnostic tools such as TVS or CA-125 biomarker have had limited success in the early detection of OC (sensitivity in stage I/II OC < 60% and overall < 88.6%) [5–7].

Where the latter diagnostic methods could not provide satisfying results, miRNAs have shown promising potential as biomarkers in various malignant diseases such as breast cancer (BC) [8–10]. Urinary miRNAs, in particular, have shown great potential in BC [11], as well as in other cancer entities like urothelial and pancreatic cancer [12, 13]. MiRNAs are small, single-stranded and non-coding RNA molecules counting approximately 20–22 nucleotides and could be detected in body fluids of healthy and diseased patients [14–16]. Incorporated into the RNA-induced silencing complex (RISC), miRNAs play a key role in specifically regulating messenger RNAs (mRNAs) posttranscriptionally [15, 17, 18]. They are involved in apoptosis, carcinogenesis, metastasis, invasion, proliferation and chemoresistance in general and in OC specifically, play an important role as oncogenes and tumor suppressor genes and are up-/down-regulated during carcinogenesis since they are binding to fragile regions on several chromosomes [15, 19–22]. Furthermore, multiple analyses have proven exosomal trafficking of miRNAs which confirms their role in cell–cell communication and therefore as possible circulating biomarkers [23, 24]. MiRNAs in contrast to proteins as biomarkers display different advantages: expression level alterations occur simultaneously to the underlying biological mechanisms, stability in most endogenous and exogenous fluids and under extreme conditions like high temperatures, long-term storage and extreme pH values like in urine [14, 23, 25]. The existing body of literature on miRNAs in OC includes OC-specific miRNAS in vitro, in tissue and circulating miRNAs. An overview of miRNA studies on OC is provided in Table 1.

**Table 1:** Overview of all included studies on miRNA expression levels in OC

Reference	Histology	Analyzed matrix	Findings
Zhang et al. [48]	EOC FIGO: all	In vitro	Upregulated: miR-26b, miR-182, miR-103, miR-26a Downregulated: miR-127, miR-134, miR-154*, miR-410, miR-377, miR-100, miR-432, miR-368, miR-154, miR-495, miR-376a, miR-323, miR-376b, miR-370, miR-299, let-7d, miR-155, miR-140, miR-222, miR-337, miR-124a, miR-99a, miR-331, miR-104, miR-150, miR-184, miR-152, miR-145, miR-424, miR-224, miR-302c
Dahiya et al. [58]	All subtypes FIGO: all	Tissue In vitro	Upregulated: miR-221, miR-146b, miR-508 Downregulated: let-7f, miR-106b, miR-134, miR-155, miR-21, miR-346, miR-422a, miR-424, miR-519a, miR-648, miR-662
Iorio et al. [38]	EOC FIGO: all	Tissue In vitro	Upregulated: miR-200a, miR-200b, miR-200c, miR-141 Downregulated: miR-140, miR-199a, miR-199b, miR-145, miR-143, miR-125a, miR-125b, miR-101, miR-212, miR-222
Yang et al. [41]	EOC FIGO: all	Tissue In vitro	Upregulated: miR-199a, miR-424, miR-302d, miR-320, miR-214, miR-200a, miR-29a Downregulated: miR-493, miR-494, miR-125b, miR-100, let-7a, let-7b, let-7c
Nam et al. [39]	SEOC FIGO: all	Tissue	Upregulated: miR-200b, miR-21, miR-200c, miR-141, miR-20a, miR-27a, miR-16, miR-93 Downregulated: miR-145, miR-125b, miR-100, miR-99a, miR-26a, miR-10b, miR-143, miR-214, let-7b, miR-29a, miR-125a
Wyman et al. [40]	All subtypes FIGO: III + IV	Tissue	Upregulated: miR-182, miR-200c, miR-142-3p, miR-200b, miR-135b, miR-200a, miR-195, miR-126*, miR-26b, miR-10b, miR-126, miR-199b-5p, miR-107, miR-30b, miR-192, miR-335, miR-32, miR-20a, miR-30c, miR-143, miR-92a, miR-199b-3p, miR-99a, miR-26a, miR-18a, miR-16, miR-15a, miR-30e, miR-194, miR-29c, miR-30d, miR-106b, Downregulated: miR-127-3p, miR-377*, miR-382, miR-493, miR-409-3p, miR-193a-5p, miR-210, miR-935, miR-100, miR-31, miR-22, miR-152, miR-379, miR-185, miR-221, miR-744, miR-21*, let-7a*, miR-574-5p, miR-31*, miR-130b, miR-149, miR-423-5p, miR-1308, miR-629, miR-320a
Calura et al. [47]	EOC FIGO: I	Tissue (histotype specificity examined)	Upregulated: miR-30a, miR-30a*, miR-192/194 cluster Downregulated: none
Taylor et al. [26]	SEOC FIGO: all	Serum	Upregulated: miR-21, miR-141, miR-200a, miR-200b, miR-200c, miR-203, miR-205, miR-214 Downregulated: none
Chung et al. [27]	SEOC FIGO: all	Serum	Upregulated: none Downregulated: miR-132, miR-26a, let-7b, miR-145
Surayawanshi et al. [28]	SEOC + others FIGO: all	Plasma	Upregulated: miR-16, miR-21, miR-191, miR-16, miR-191, miR-4284 Downregulated: none
Resnick et al. [29]	SEOC + others FIGO: all	Serum	Upregulated: miR-21, miR92, miR-93, miR-126, miR-29a Downregulated: miR-155, miR-127, miR-99b
Häusler et al. [30]	SEOC + others Recurrent disease	Whole blood	Upregulated: miR-30c-1 Downregulated: miR-342-3p, miR-181a, miR-450-5p
Zheng et al. [55]	All subtypes FIGO: all	Plasma	Upregulated: miR-205 Downregulated: let-7f
Meng et al. [53]	SEOC + others FIGO: all	Serum	Upregulated: miR-7, miR-429 Downregulated: miR-25, miR-93
Kapetanakis et al. [52]	All subtypes FIGO: all		Upregulated: miR-200b Downregulated: none
Kan et al. [51]	SEOC FIGO: III + IV	Serum	Upregulated: miR-182, miR-200a, miR-200b, miR-200c Downregulated: none
Shapira et al. [54]	SEOC FIGO: all	Plasma	Upregulated: miR-1274a, miR-625-3p, miR-720 Downregulated: miR-106b, miR-126, miR-150, miR-17, miR-20a, miR-92a
Zuberi et al. [32]	SEOC FIGO: all	Serum	Upregulated: miR-200a, miR-200b, miR-200c Downregulated: none
Gao et al. [31]	All subtypes FIGO: all	Serum	Upregulated: miR-141, miR-200c Downregulated: none
Liang et al. [34]	All subtypes FIGO: all	Serum	Upregulated: none Downregulated: miR-145
Hong et al. [33]	SEOC + others FIGO: all	Serum	Upregulated: miR-221 Downregulated: none
Zhou et al. [35]	SEOC FIGO: all	Urine	Upregulated: miR-30-5p Downregulated: 37 different miRNAs
Zavesky et al. [36]	All subtypes FIGO: all	Urine	Upregulated: miR-92a, miR-200b Downregulated: miR-106b, miR-100

*EOC* epithelial ovarian cancer, *SEOC* serous epithelial ovarian cancer, *FIGO* Fédération Internationale de Gynécologie et d'Obstétrique

### OC-specific circulating microRNAs

Upcoming approaches target the investigation of disease specific miRNA expression profiles in human body fluids such as blood and urine aiming at an early diagnosis. The comparison of tissue and serum miRNA expression levels revealed a definite relationship between tissue miRNAs and tumor-derived miRNAs in human body fluids [26]. Mir-132, miR-26a, let-7b and mir-145 showed promising potential as novel biomarkers in serous EOC, since they exhibited to be down-regulated in serum specimens [27]. Surayawanshi et al. also compared tissue and plasma miRNA expression profiles using global profiling. They concluded that different expression profiles in both media might account for another origin of plasma miRNAs than the ovarian malignancy [28].

Resnick et al. applied qRT-PCR to compare miRNA expression profiles of 21 miRNAs in 28 EOC patients and 19 HCs [29]. Five miRNAs were upregulated and three miRNAs were down-regulated in the serum of EOC patients (see Table 1) [29]. Another study on whole blood OC samples of 24 OC patients with recurrent disease revealed the deregulation of 147 miRNAs in OC patients compared to HCs, with miR-30c1 upregulated and miR-342-3p, miR-181a and miR-450b-5p down-regulated [30]. Further blood-based studies on miRNA expression profiles in OC comprise the studies by Zheng et al., Meng et. al., Kapetanakis et al., Kan et al. and Shapira et al.. For detailed information, see Table 1.

The miRNA-200-family underwent extensive research as diagnostic biomarker in OC. MiR-200a, miR-200b, miR-200c and miR-141 were found upregulated in the serum of OC patients in two independent studies and showed significant correlation with prognosis, tumor stage and histology [31, 32]. MiR-145 as well as miR-221 showed promising potential in the serum-based discrimination OC patients from HCs [33, 34].

Zhou et al. investigated exosomal miRNA expression in urine samples of 39 serous EOC patients pre- and postsurgically, 26 patients presenting with another gynecological disease and 30 HCs applying qRT-PCR [35]. First, they found miR-30a-5p upregulated as well as 37 miRNAs down-regulated comparing presurgical OC and HC samples. Second, stratifying for stage and metastatic status, they showed a distinct association between miR-30a-5p and early-stage OC as well as lymph node metastasis. Third, in urine samples of gastric and colon cancer patients miR-30a-5p showed to be down-regulated, supporting its OC specificity. And finally, in postsurgical OC samples of the same patients, the expression levels of miR-30a-5p were clearly lower than presurgically suggesting OC strongly as its origin [35]. Interestingly, miR-30a-5p was upregulated in the supernatant of OC cell lines, which displays a possible excretion mechanism. They also performed a knockout of miR-30a-5p resulting in a significant decrease of OC cell proliferation as well as migration [35].

Zavesky et al. analyzed cell-free urine of eleven OC as well as endometrial cancer patients [36]. They compared not only pre-and postsurgical specimen of the same patients, but also contrasted them to three HCs. Among the 18 included miRNAs, miR-92a, miR-200b, miR-106b and miR-100 exhibited a significant deregulation comparing OC and HC. Mir-92a and mir-200b were upregulated, while miR-100 and miR-106b were down-regulated [36].

## Methods

### Cohorts and sampling

After the positive ethical vote (Number 36/12 and 386/16 approved by the Institutional Ethical Review Board of the University of Freiburg) as well as positive informed consent each patient included into the study provided an indefinite volume of urine. In the presented case–control study, thirteen patients and 17 HCs are included for comparative analysis. The urine samples were collected at the Department of Gynecology and Obstetrics of the University Medical Center Freiburg (ten cancer samples, 17 HCs) between January 2015 and May 2016 and of the University Clinic of Bergen (Norway) (three cancer samples). Inclusion criterion for the disease group was newly diagnosed primary OC (FIGO I–IV), whereas for the control group, a detailed gynecological examination without evidence of any gynecologic malignancy prior to probes collection was necessary. Exclusion criteria were previous cancer diseases, simultaneous malignancies, chemotherapy or radio-chemotherapy prior to sample collection, autoimmune diseases, diabetes mellitus type 1 and infections. OC and HC specimen were collected simultaneously and age matched (see Table 2).

**Table 2:** Ovarian cancer patients included into the study

Sample no.	Age	Histologic subtype	Stage	TNM	Grading	Confounding diagnosis
1	47	Serous Adenocarcinoma	FIGO IIIc	pT3c, pN1, cM0	G3	None
2	53	Serous Adenocarcinoma	FIGO IIIc	pT2a, pN1, cM0	G3	Zoeliakie
3	56	Serous Adenocarcinoma	FIGO IIIc	pT3c, pN1, cM0	G3	None
4	76	Serous Adenocarcinoma	FIGO IV	pT3c, pN1, cM0	G3	Gastritis
5	48	Serous Adenocarcinoma	FIGO IIIc	pT3a, pN1, cM0	G3	Hypothyreosis
6	79	Serous Adenocarcinoma	FIGO IV	pT3c, pN1, cM0	G3	None
7	65	Serous Adenocarcinoma	FIGO IIIc	pT3c, pN1, cM0	G2	Esophagitis, bulbitis, gastritis
8	24	Sertoli-Leydig-Cell Tumor	FIGO Ia	pT1a, pN0, cM0	–	None
9	63	Serous Adenocarcinoma	FIGO IIa2	pT3a, pN1, cM1	G3	None
10	71	Serous Adenocarcinoma	FIGO IIIc	pT3c, pN1, cM0	G3	None
11	–	Serous Adenocarcinoma	–	–	–	–
12	–	Serous Adenocarcinoma	–	–	–	–
13	–	Serous Adenocarcinoma	–	–	–	–

*TNM* tumorstatus identified by pathologist, *G* grading, *FIGO* tumorstatus according to the French organization Fédération Internationale de Gynécologie et d'Obstétrique (FIGO)

All urine samples were collected in 100 ml sterile lockable urine collection cups (Sarstedt, Germany). After the urine acquisition, all samples were stored at − 80 °C until further processing. Prior to the final analysis, extensive centrifugation at 4,000 rpm for five minutes was performed.

### Cell culture

The established human tumor cell lines, SK-OV-3 and EFO-27 and OAW-42 were cultured in a humidified incubator (37 °C, 5% CO_2_, 95% air) and maintained according to the recommended cell line-specific culturing conditions. Cells were transferred into 25 cm^2^ cell culture flasks (Greiner Bio-One, Frickenhausen, Germany) until they reached 70% confluency. For hypoxia experiments, cells were placed in a hypoxic chamber (3% O_2_; mentioned as hypoxia). For acidosis experiments, culture media were supplemented with 2-Hydroxypropionic acid (Carl Roth, Karsruhe, Germany 0.2%, pH 6.2). Cells were cultured in parallel experiments under normal conditions (used as control). All treatments lasted for 24 h. Triplicates were generated. Afterwards, cells and cell culture media were processed separately. Cells underwent direct lysis according to the RNA isolation protocol, whereas cell culture media underwent extensive centrifugation (4000 rpm for ten minutes) before further processing.

### RNA isolation, reverse transcription, poly-A-tailing and pre-amplification

Two RNA isolation protocols were used: analytic Jena´s innuPREP Micro RNA Kit (Analytic Jena, Jena, Germany) for the cells and Exiqon´s miRCURY RNA Isolation Kit—Biofluids (Exiqon, Qiagen GmbH, Hilden, Germany) for the liquid specimen. Before performing Exiqon´s miRCURY RNA Isolation Kit—Biofluids, extensive centrifugation steps eradicated all cellular material and separated DNA from RNA. The NanoDrop^®^-ND-1000-Spectrophotometer (NanoDrop Technologies, Wilmington, DE, USA) served to determine the RNA concentration spectrometrically. The isolated RNA was finally stored at − 20 °C until further processing.

Subsequently, a reverse transcription (RT) protocol was performed (miScript Reverse Transcription Kit, Qiagen GmbH, Hilden, Germany) that generated cDNA of miRNAs only.

### Quantitative real-time PCR

Quantitative real-time polymerase chain reaction (qPCR) served as detection method for determining miRNA expression levels in cell culture, cell culture media and urine samples. We processed qPCR on the Eppendorf 480 Mastercycler (Eppendorf, Hamburg, Germany). Duplicates of each sample were analyzed. For the qPCR, one µl cDNA and nine µl of an in-house qPCR mastermix (containing TRIS pH 8.1, dATP, dCTP, dGTP, dTTP, magnesium, potassium ammonium, SYBRGreen (Jena Bioscience, Jena, Germany), enhancers, HotStart Taq Polymerase (Jena Bioscience)) were used. A negative control (ten µl mastermix, no cDNA) and a RT (no RNA for reverse transcription, 1 µl unspecific cDNA, 9 µl mastermix) were added, to evaluate if specific or unspecific products were amplified. For primer sequences, see Table 3.

**Table 3:** List of primers used for qPCR

Universal antisense	5′-GAA CAG TAT GTG TCA CAG ACG TAC-3′
Let-7a	5′-GCGG TGAGGTAGTAGGTTGTAT-3′
Let-7d	5′-GCGG AGAGGTAGTAGGTTGCATA-3′
miR-10a	5′-GCATG TAC CCT GTA GAT CCG A-3′
miR-15a	5′-GCGG TAGCAGCACATAATGGTT-3′
miR-15b	5′-CATG CAT AGC AGC ACA TCA TG -3′
miR-19b	5′-CATG TGT GCA AAT CCA TGC A -3′
miR-20a	5′-GCGG TAAAGTGCTTATAGTGCAG-3′
miR-21	5′-GCATGCA TAG CTT ATC AGA CTG -3′
miR-25	5′ -TCA TTG CAC TTG TCT CGG T -3´
miR-100	5′-GCATT AAC CCG TAG ATC CGA-3′
miR-103	5′-CGG AGCAGCATTGTACAGG-3′
miR-125b	5′-GCAT TCC CTG AGA CCC TAA C-3′
miR-155	5′-GCATGCA TTA ATG CTA ATC GTG A -3′
miR-191	5′-GCGG CGG AAT CCC AAA AGC AG-3′
miR-222	5′-GCATG CTCAGTAGCCAGTGTAG-3′

The part of capital letters represents the miRNA-specific primer, the uncapitalized part represents the melting temperature overhang

### Analysis and statistics

We applied a multivariable linear regression model to the log-transformed expression values with cell line (SK-OV-3, EFO-27, OAW-42) and its two-way and three-way interactions with treatment (control, hypoxia, acidosis) and compartment (intra-/extracellularly) as independent variables. All statistical methods involve Δ*C*_t_ values normalized against the mean value of miR-25, -103 and -191. To calculate the influence of hypoxia and acidosis Δ*C*_t_ values of treated and untreated probes were compared statistically which is represented in the relative expression (= 2^−ΔCT^).

For the interpretation of the multivariable analysis, all miRNA expression levels are compared to the intercept. The intercept represents cell line EFO-27 under control condition in the intracellular compartment. All statistical results with a *p* value of 0.05 or lower were interpreted as significant.

## Results

Summarizing, all analyzed miRNAs except miR-155 could be detected intra- and extracellularly. MiR-155 was detectable extracellularly only, however, to an extremely low degree (Δ*C*_t_ values between 22.34 and 29.20). Additionally, miR-21 did not show any statistically significant results neither on the intra- nor extracellular level under any analyzed condition in neither of the cell lines. The following paragraph pictures relevant findings only. All expression level regulations of miRNAs must be interpreted in comparison to the expression level of the same miRNA in the arbitrary reference (EFO-27, untreated, intracellularly). For a full list of results as well as the expression levels of each miRNA for the intercept and its confidence intervals, see supplemental data 1–3.

### Intracellular expression level alterations

**Fig. 1:**
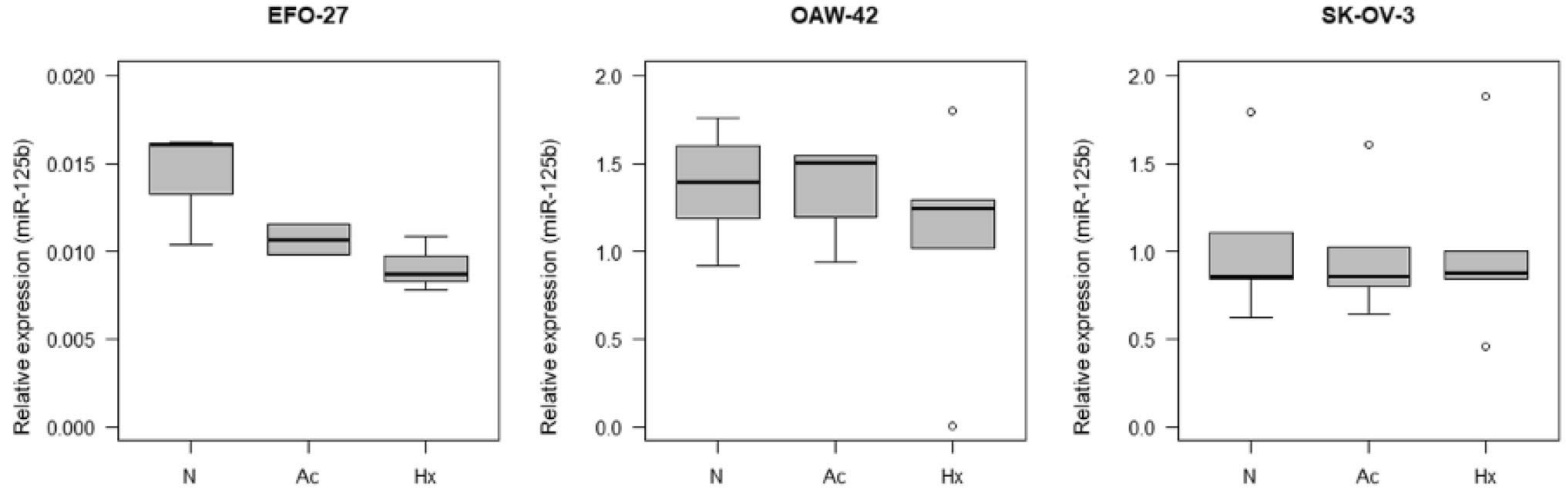
Intracellular relative expression of miR-125b in EFO-27, OAW-42 and SK-OV-3 cells. *N* control, *Ac* acidosis, *Hx* hypoxia

**Fig. 2:**
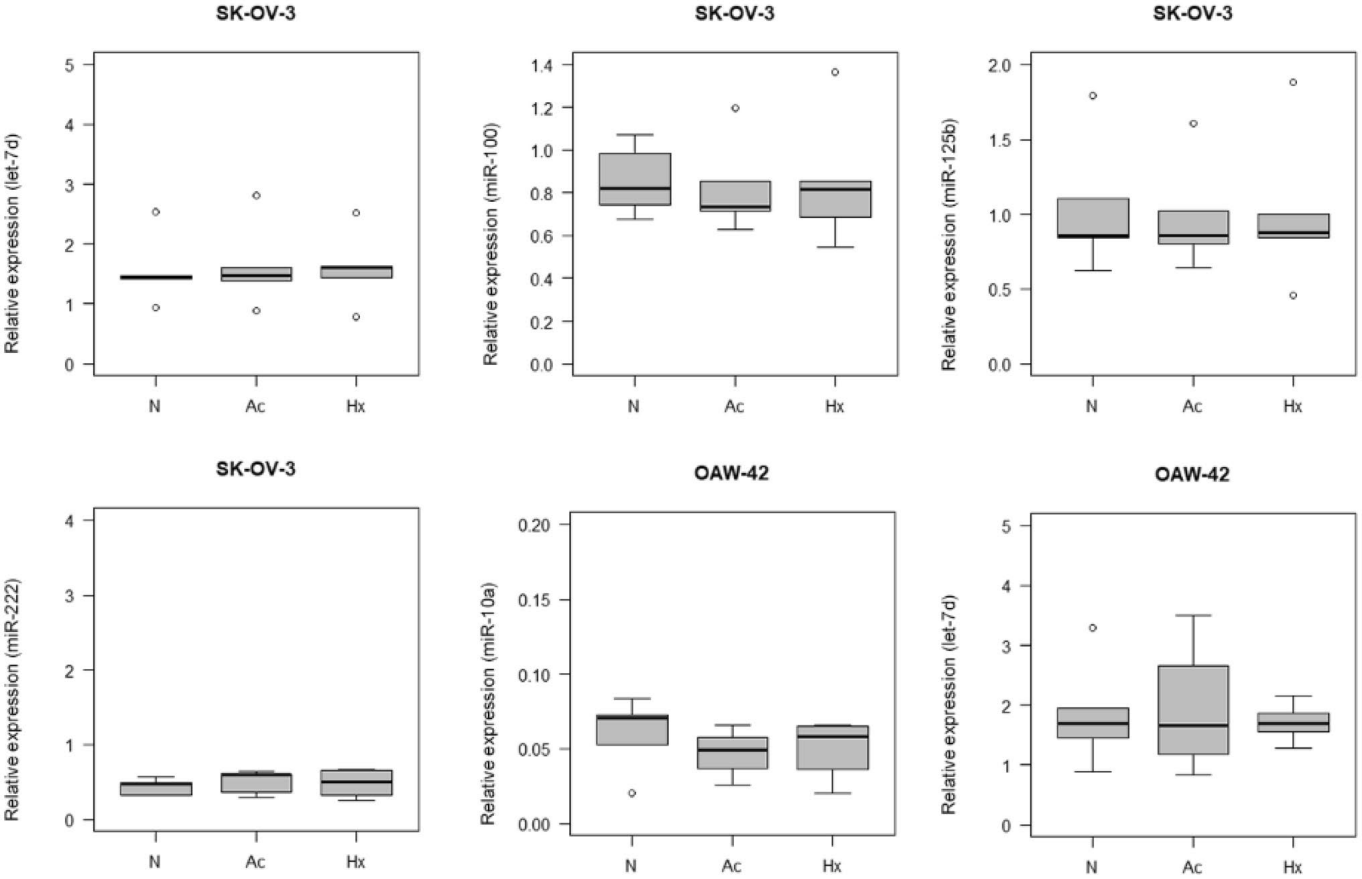
Intracellular relative expression of let-7d, miR-100, miR-125b and miR-222 in SK-OV-3 cells and of let-7d and miR-10a in OAW-42 cells. *N* control, *Ac* acidosis, *Hx* hypoxia

Focusing on intracellular expression levels only, miR-125b expression levels showed significant alterations in all analyzed cell lines. Expression levels were higher in SK-OV-3 (70.14; CI 10.71–459.31; *p* < 0.001) and OAW-42 (95.99; CI 14.66–628.61; *p* < 0.001) cells, compared to EFO-27 (see Fig. 1). Furthermore, in SK-OV-3, let-7d (0.35; CI 0.15–0.83; *p* = 0.02) and miR-222 (0.16; CI 0.05–0.53; *p* = 0.004) showed lower expression levels, miR-100 (10.29; CI 2.17–48.83; *p* = 0.005) showed higher expression levels compared to EFO-27. Additionally, miR-10a (0.06; CI 0.01–0.44; *p* = 0.008) and let-7d (0.40; CI 0.17–0.95; *p* = 0.041) showed lower intracellular expression levels in OAW-42 cells (see Fig. 2).

### Extracellular expression level alterations

**Fig. 3:**
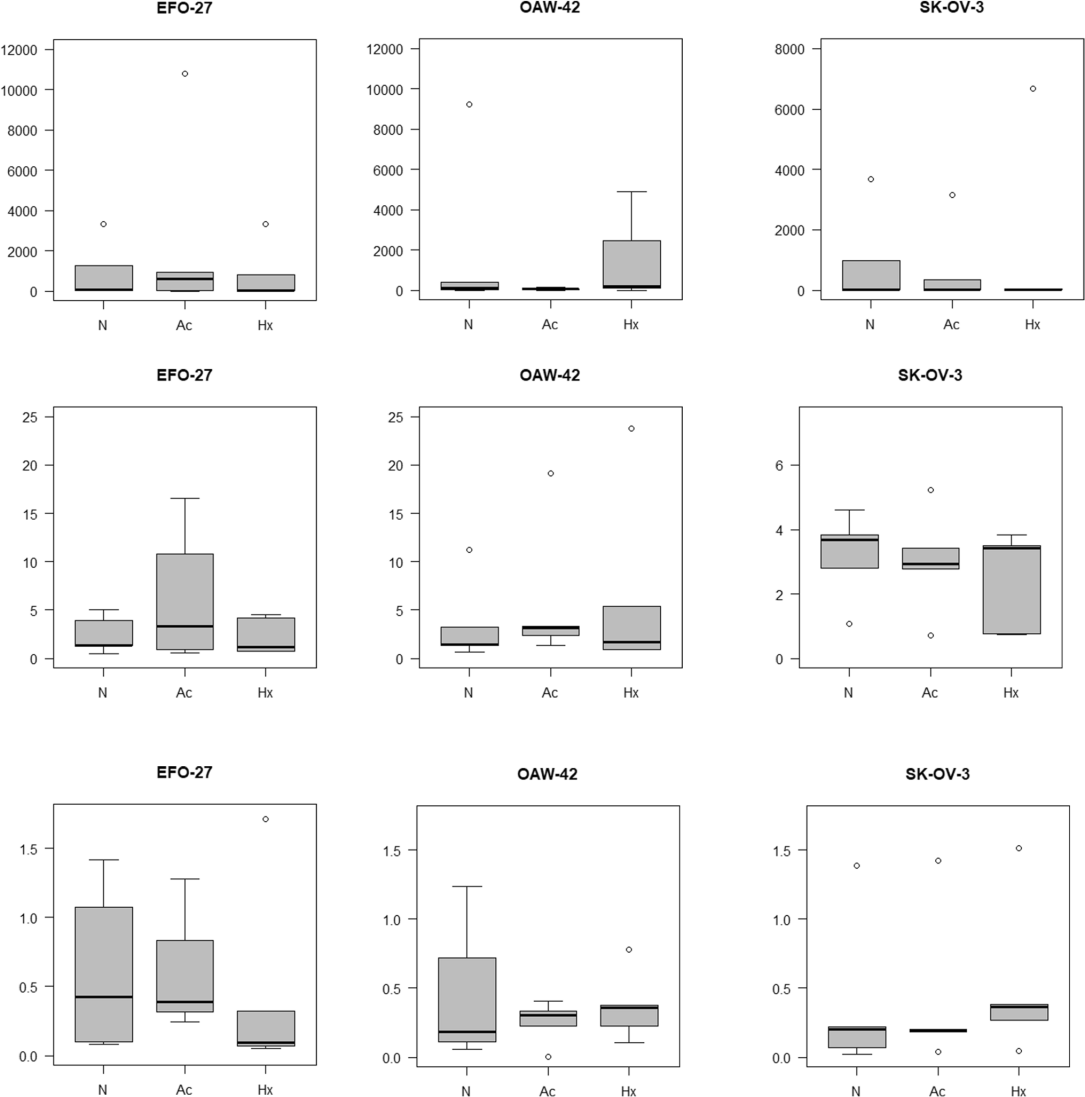
Extracellular expression alterations: miR-15a, miR-20a and miR-222 in EFO-24, OAW-42 and SK-OV-3 cells. *N* control, *Ac* acidosis, *Hx* hypoxia

Extracellularly, compared to the intercept, miR-15a and miR-20a showed altered expression levels in the analyzed OC cell lines, whereas they were stable in the intracellular compartment. MiR-15a (SK-OV-3: 536.59; CI: 29.26–9838.90; *p* < 0.001; EFO-27: 428.64; CI 23.38–7859.65; *p* < 0.001; OAW-42: 438.58; CI 23.92–8041.82; *p* < 0.001) and miR-20a (SK-OV-3: 5.08; CI 1.84–14.00; *p* = 0.002; EFO-27: 3.73; CI 1.35–10.28; *p* = 0.013; OAW-42: 5.74; CI 2.08–15.83; *p* = 0.001) showed higher expression levels. In EFO-27 and OAW-42, miR-222 showed lower expression levels (EFO: 0.12; CI 0.03–0.42; *p* = 0.002; OAW-42: 0.14; CI 0.04–0.48; *p* = 0.003) (see Fig. 3 and supplemental data for box plots, expression levels and confidence intervals). Additionally, there were expression level alterations in the extracellular compartment of the EFO-27 cell line compared to the reference: let-7a (0.11; CI 0.03–0.40; *p* = 0.001), let-7d (0.18; CI 0.07–0.42; *p* < 0.001), miR-15b (0.48; 0.23–1.02; *p* = 0.060) and miR-125b (6.05; CI 0.92–39.61; *p* = 0.065) and expressed lower, whereas miR-15a, miR-20a (see above) and miR-19b (3.15; CI 0.95–10.44; *p* = 0.065) expressed higher. In OAW-42 cells supernatant, let-7a (0.26; CI 0.07–0.97; *p* = 0.048) expressed lower compared to the intercept.

### Expression level alterations under hypoxia and acidosis

**Fig. 4:**
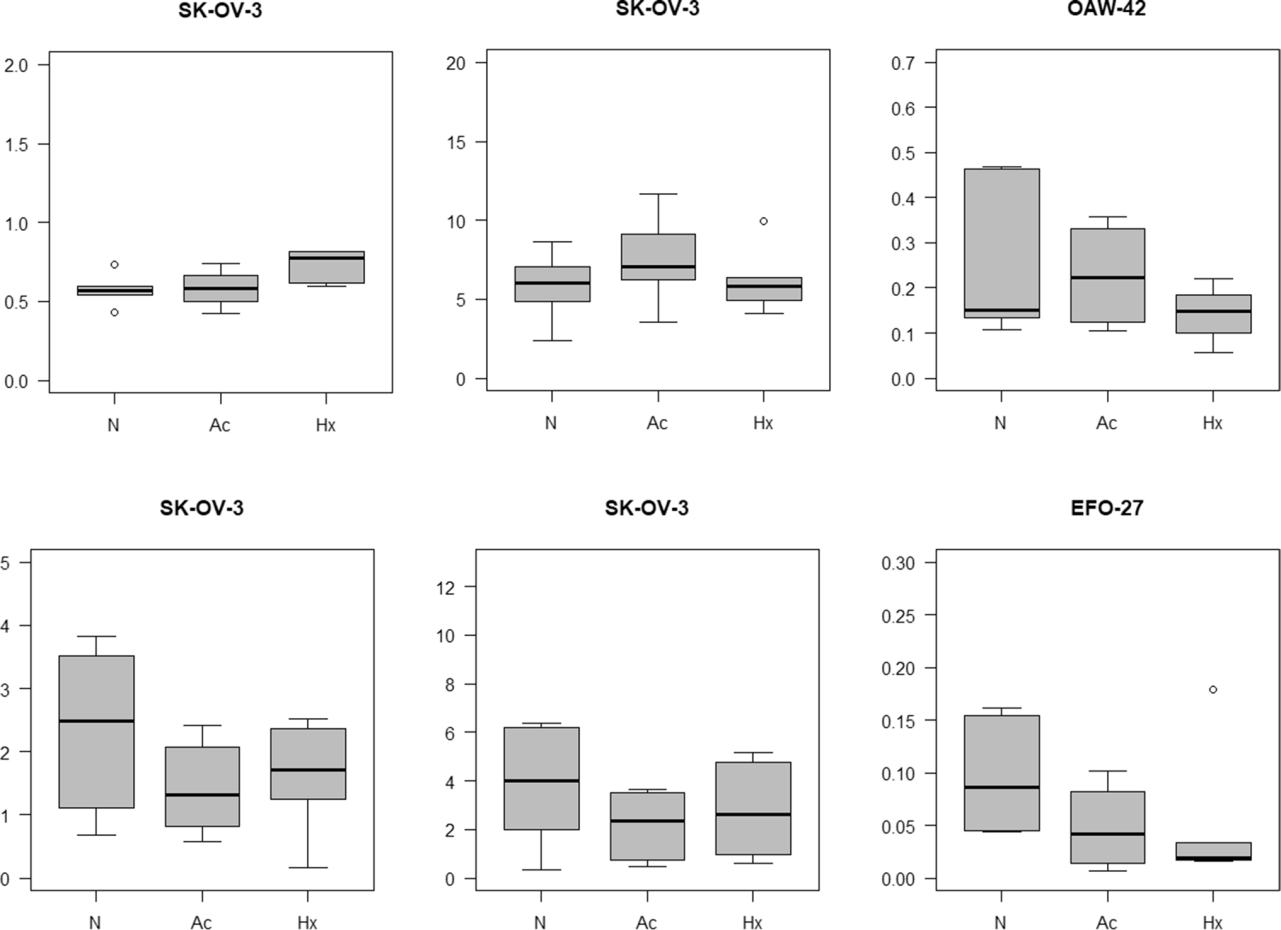
Regulated miRs under hypoxia and acidosis. *N* control, *Ac* acidosis, *Hx* hypoxia

**Fig. 5:**
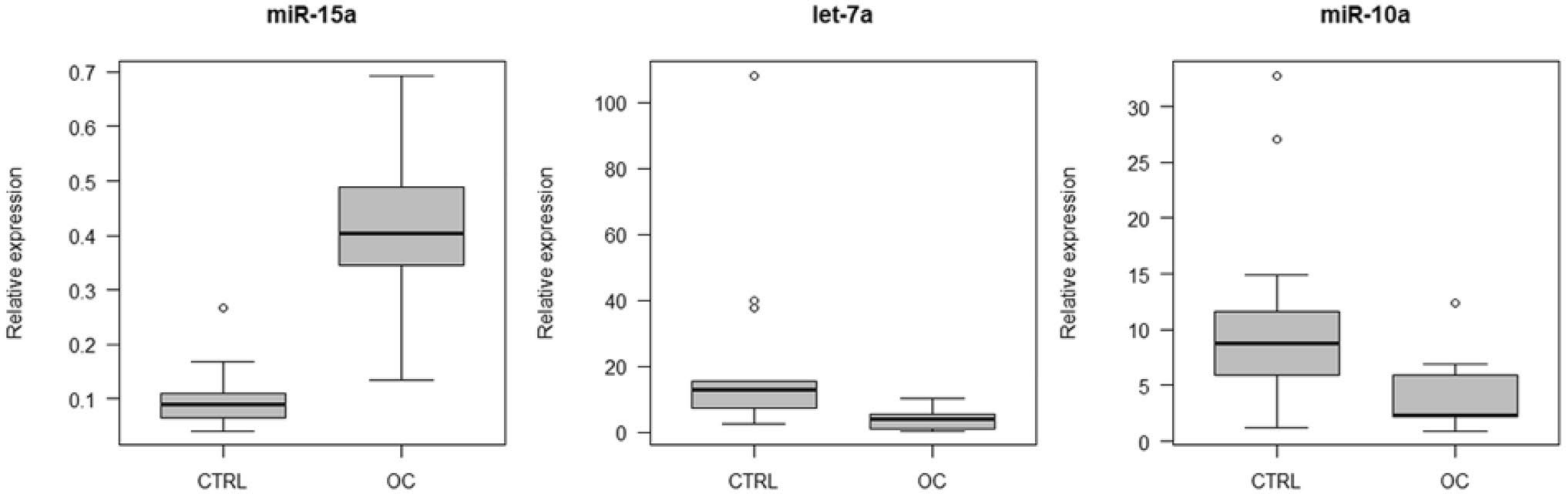
Deregulated miRNAs in the urine of OC patients compared to HC. Relative expression is shown. *CTRL* healthy controls, *OC* ovarian cancer patients

Regarding hypoxia and acidosis, no significant miRNA alterations were visible after the application of the multivariable linear regression model. Still, the raw data and the boxplots revealed some promising miRs: in the intracellular compartment of SK-OV-3 cells, hypoxia led to an upregulation of miR-20a while acidosis led to an upregulation of miR-21. Inside OAW-42 cells, acidosis led to an upregulation of miR-19b (see Fig. 5). In EFO-27 cells, no alterations occurred. In the extracellular compartment of the SK-OV-3 cell line, hypoxia led to a downregulation of let-7d. Simultaneously, acidosis led to an even stronger downregulation of miRNAs let-7a and let-7d. In the OAW-42 cell line, neither hypoxia nor acidosis led to a significant alteration of the same miRNAs. In the extracellular compartment of the EFO-27 cell line, acidosis triggered a downregulation of miR-125b (see Fig. 4; for all changes caused by hypoxia and acidosis see supplemental data 1–3).

### Urinary results

Finally, the expression level of miR-15a was higher in the urine of OC patients than in the urine of HCs (*p* = 0.0319) whereas the expression level of let-7a was lower in OC patients (*p* = 0.0199). MiR-10a tended to be slightly down-regulated in the urine of OC patients (*p* = 0.0571) (see Fig. 5). MiRNAs let-7d, miR-15b, miR-19b, miR-20a, miR-21, miR-100, miR-125b and miR-222 did not show significant expression level alterations and miR-155 was not detectable.

## Discussion

The major source of existing discrepancies across studies lies in differing methodological approaches, a lack of suitable housekeeper miRNAs, disease and tumor heterogeneity as well as in the nature of miRNAs themselves.

The methodological approach and single steps of miRNA analysis have a significant impact on the final results [23, 37]. For example, northern blotting, microarray-based detection, next-generation sequencing and real-time RT-PCR differ in sensitivity and specificity and their random use has led to inconsistent results.

Dahiya et al. describe this issue in their study and literature research [17]. MiRs-21, -155 and let-7d were down-regulated in tissue and cell lines, whereas miR-100 showed to be upregulated in that microarray-based approach. In addition, they found that only 16 of 192 analyzed miRNAs showed consistent expression patterns across studies [17]. The given study demonstrated a downregulation of let-7d in cell culture analyses intra- and extracellularly and found higher expression levels of miR-100 in the intracellular compartment of SK-OV-3 cells only.

Four more studies on OC tissue showed diverging results as well [38–41]. Iorio et al. applied microarray analyses and found different miRNAs to be deregulated [38]. MiR-125b1 was down-regulated. MiR-100 showed lower expression levels in OC tissues compared to HCs [38]. Nam et al. showed an upregulation of miR-20a and miR-21 in ovarian tumor tissue [39]. In the presented study, miR-125b showed a similar lower expression in EFO-27 intracellularly, extracellularly and tendentially under acidosis. In SK-OV-3 and OAW-42 cell lines, it showed higher expression levels intracellularly only. This is why it must be hypothesized that miR-125b is subtype specific. Moreover, miR-100 exhibited higher expression levels intracellularly in SK-OV-3 and miR-20a showed higher expression levels in the supernatant of all three analyzed cell lines in our study as well.

Methodologically, Wyman et al. used next-generation sequencing and subsequent qRT-PCR [40]. They found miR-15a and miR-20a to be upregulated like proven extracellularly in the given study. However, they also showed a downregulation of miR-21 and miR-100 [40].

Regarding the impact of hypoxia and acidosis on miRNA expression levels in OC, results are scarce. We found sporadic alterations of miRNA expression levels as well (see results). Giannakakis et al. demonstrated the involvement of miR-210 in the HIF pathway [42]. Compared to our study, hypoxia was methodologically induced using a lower amount of oxygen (1.5% vs. 3%) and a different panel of miRNAs was analyzed [42]. Another study on endometrial cancer cell lines also showed sporadic alterations only: miR-15a, miR-20a, miR-20b, miR-21 under hypoxia and let-7a, miR-22 and miR-125b under acidosis [43]. These results were inconsistent with comparable studies [44].

We hypothesize that OC patients can be distinguished from HC comparing their urinary miRNA expression levels. However, urine as well as cell culture supernatant are both challenging for cancer detection because of their biochemical conditions. The feasibility of urinary miRNA-based detection of cancer has already been proven in other tumor entities like BC [11]. Several studies verified the stability of miRNAs under harsh conditions like extreme pH values [23]. Compared to proteins, miRNAs undergo less degradation through ribonucleases due to their packaging into exosomes and the RISC and also because of their small size [14, 30]. However, the amount of total miRNA in urine and cell culture supernatant is small, which emphasizes that miRNA quantity and quality are crucial for the final detection [25, 45].

Targeting the correlation of tumor cell-derived and urinary miRNAs, one study on OC tissue and serum of the same patients conducted by Taylor et al. found matching expression patterns of eight miRNAs and, therefore, hypothesized that miRNAs in the serum derive directly from the tumor itself [26]. Nakamura et al. also suggest that miRNAs in biofluids reflect tissue miRNA expression levels accurately [23]. Microarray analyses of tissue, ascites and serum of EOC patients also showed uniform alterations [27]. However, in serum, additional miRNAs evolved to be regulated [27]. In our study, we detected differing miRNA signatures in cell culture, in cell culture supernatant and in urine.

Furthermore, OC detection based on urinary miRNAs is hindered by disease heterogeneity. OC is a highly individual disease that differs in histology, stage, metastatic status and molecular tumor characteristics [46]. The studies conducted by Iorio et al. and Calura et al. picture this as well [38, 47]. Both showed histotype-specific miRNA expression signatures. MiR-222 showed specific alteration in endometrioid and clear cell subtypes, whereas miR-21 did in endometrioid subtypes only [38, 47]. In our in vitro study, we were also able to detect subtype specific miRNA alterations. Regarding the patient samples collected in this study, it is important to emphasize the inclusion of one sample of Sertoli–Leydig cell tumor. This also reflects tumor and disease heterogeneity but the statistical significance achieved despite the inclusion indicates the robustness of the found miRNAs and suggests, that the detected miRNA alterations are ovarian cancer specific and not only subtype specific for serous adenocarcinomas.

Not only disease but also tumor heterogeneity complicate miRNA-based OC detection. Additionally, the tumor microenvironment consists of different cells and acellular components connected to different miRNA alterations. According to this, the same tumor might be able to exhibit a varying miRNA expression pattern at different states of its existence. This could explain the inconsistency of miRNA alterations across the published studies.

For example, miR-15a as well as let-7d showed significant downregulation in a cell culture study conducted by Zhang et al. [48]. Interestingly, the degree of downregulation rose with stage and was not detectable in 23.9% of the examined samples. They finally suggested these two miRNAs to be tumor suppressors [48]. MiR-15a was neither down- nor upregulated in the intracellular compartment of the given study but was significantly elevated in the extracellular compartment of all three analyzed cell lines. This suggests that the intracellular downregulation or absence of miR-15a is a possible result of an upregulated trafficking into the tumor microenvironment.

Moreover, the significant downregulation of let-7d in OC cell lines could be proven intracellularly in SK-OV-3 and OAW-42, extracellularly in EFO-27 and OAW-42 and under hypoxia and acidosis in SK-OV-3. Let-7d emerged its role as tumor suppressor by negatively regulating the RAS-pathway in lung cancer [49]. In conjunction with tumor heterogeneity, tumor immunology revealed a crucial role in the development and formation of cancer specific miRNA patterns [50].

Resnick et al. identified miR-21 and miR-155 as possible biomarkers [29]. While miR-21 was upregulated, miR-155 was down-regulated [29]. We observed this in our in vitro study as well, but not in the urine of OC patients. This suggests that urinary miRNAs express differently because of the activity of urine-specific enzymes and several cellular mechanisms. Some other blood-based studies emphasize this hypothesis showing aberrantly altered miRNAs compared to the given urine-based study [30, 51–55]. However, it must be considered that the analyzed OC histologic subtypes and samples as well as the applied methods and the investigated miRNA panels varied tremendously across these six studies.

Finally, patient heterogeneity modifies the results of the given study to an unknown extent, given that each individual presents with many exogeneous as well as endogenous confounders. In detail, cardiovascular, rheumatologic, dermatologic, neurologic, renal and many other diseases lead to specific miRNA expression alterations [25, 56, 57].

Zhou et al. performed qRT-PCR to determine exosomal miRNA expression levels [35]. Not only OC samples but also benign ovarian tumors and gastric as well as colon carcinomas were analyzed and prove OC specificity of miR-30a-5p upregulation and the downregulation of 37 more miRNAs in OC exclusively [35].

Zavesky et al. performed qRT-PCR on cell-free urine samples as well and found miR-100 to be down-regulated [36]. The strength of this work was prior assessment of RNA quality and quantity, while the inclusion of carcinomas of the fallopian tube into the study group of only 11 patients is questionable [36].

## Conclusion

The landscape of miRNA studies in OC emphasizes their great potential for the detection of OC. With the given study we were able to demonstrate the feasibility of distinct miRNA-based discrimination of OC and HC in urine thanks to a specific miRNA signature. We were also able to widen the panel of miRs that potentially serve as diagnostic urinary biomarker in the detection of OC. However, our study also shows that urinary miRNA expression levels are massively dependent on methodological procedures. Comparing the few previous urine-based studies and the given study, there are several differences in the reported miRNA alterations in OC patients. This mirrors the great necessity of standardized miRNA extraction and detection protocols. Furthermore, to the best of our knowledge this study is the second one to demonstrate the feasibility to detect OC-specific miRNAs in cell culture supernatant and the first to prove the traceability of single miRNAs from the intracellular to the extracellular compartment and finally to urine. MiR-15a was upregulated in OC cell culture supernatant as well as in urine of OC patients which strengthens its OC-specific diagnostic potential observed in various studies before. As this study examines a small number of samples only, future studies are crucial to verify these observations.

## Supplementary Information

Below is the link to the electronic supplementary material.

Supplementary file 1 (Additional file 1.docx): Box plots. Shows box plots of relative expression levels of all analyzed miRNAs in the cell lines EFO-27, OAW-42 and SK-OV-3, in both compartments (intra (A)- and extracellular (B)) and under all analyzed treatments (untreated (N), hypoxia (Hx) and acidosis (Ac)) as well as box plots of all analyzed miRNAs in OC patients (OC) compared to healthy controls (CTRL) (DOCX 37098 KB)

Supplementary file 2 (Additional file 2.csv): Raw data. Shows ΔCt values of five cell culture sets (1–5) intra- and extracellularly of the three cell lines EFO-27, OAW-42 and SK-OV-3 under all analyzed treatments (untreated (N), hypoxia (Hx) and acidosis (Ac)) (CSV 11 KB)

Supplementary Additional file 3 (Additional file 3.txt): Results generated by R. Shows relative expression levels, confidence intervals and p values of each cell line after the statistical analysis generated by R (TXT 15 KB)

## Data Availability

All data generated or analyzed during this study are included in this published article and its supplementary information files. Additional information is available from the corresponding author on reasonable request.

## Abbreviations

BC: Breast cancer
cDNA: Complementary desoxyribonucleic acid
EOC: Epithelial ovarian cancer
FIGO: Fédération international de gynecology et obstetrique
HC: Healthy controls
HIF: Hypoxia-inducible factor
miRNA, miR: MicroRNA
mRNA: Messenger RNA
nt: Nano tons
nm: Nano meters
OC: Ovarian cancer
PCR: Polymerase chain reaction
pre-miRNA: Premature-microRNA
pri-miRNA: Primary-microRNA
RISC: RNA-induced silencing complex
RNA: Ribonucleic acid
RT: Reverse transcription
SEOC: Serous epithelial ovarian cancer
tRNA: Transfer ribonucleic acid
UTR: Untranslated ending
WHO: World health organization
