# Supplementary material for: Evaluation of circulating microRNAs as non-invasive biomarkers in the diagnosis of ovarian cancer: a case–control study
Source: Arch Gynecol Obstet. 2021 Dec 10;306(1):151–63. doi: 10.1007/s00404-021-06287-1 (PMC9300512; doi:10.1007/s00404-021-06287-1)
Supplement: Supplementary file 1 — (Additional file 1.docx): Box plots. Shows box plots of relative expression levels of all analyzed miRNAs in the cell lines EFO-27, OAW-42 and SK-OV-3, in both compartments (intra (A)- and extracellular (B)) and under all analyzed treatments (untreated (N), hypoxia (Hx) and acidosis (Ac)) as well as box plots of all analyzed miRNAs in OC patients (OC) compared to healthy controls (CTRL) (DOCX 37098 KB) [file 404_2021_6287_MOESM1_ESM.docx]

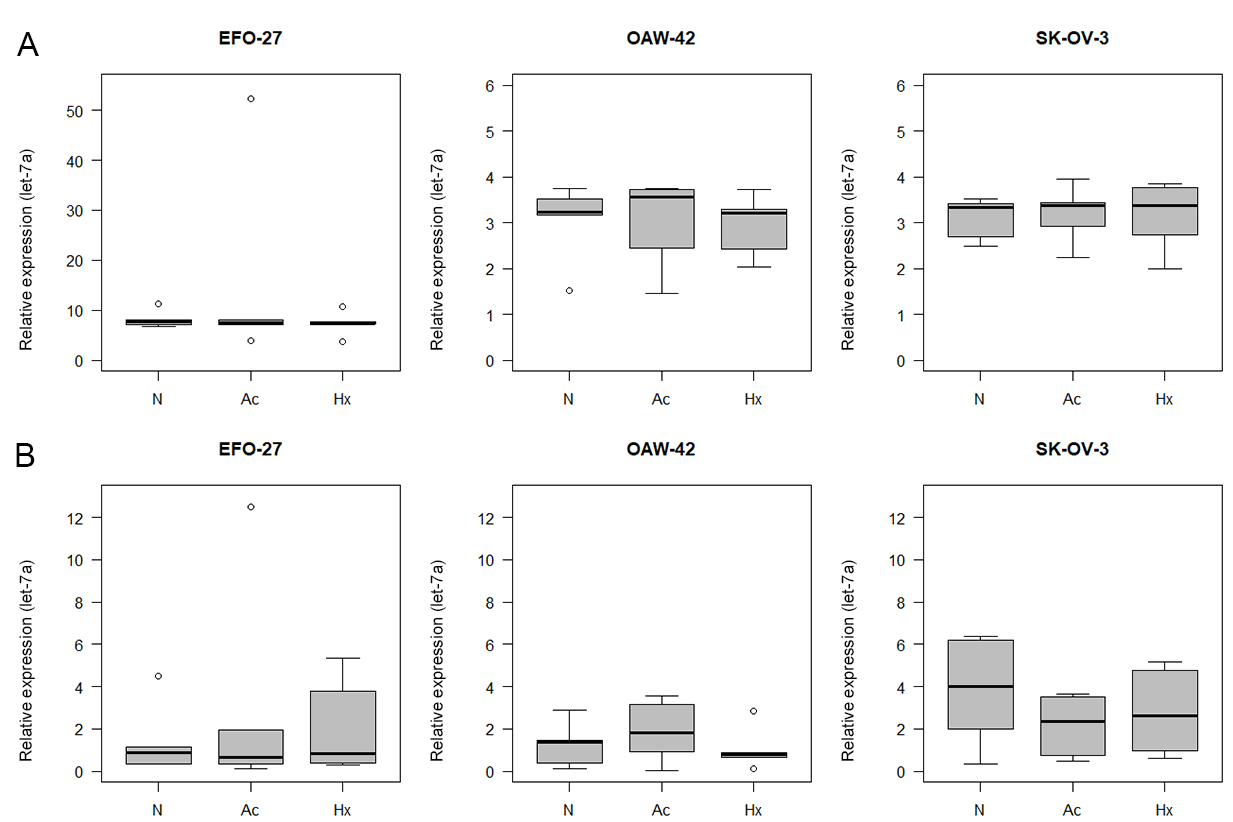


**Supplemental Figure 1:** Expression levels of let-7a intra- (A) and extracellular (B) in the three analyzed cell-lines EFO-27, OAW-42 and SK-OV-3 under normal conditions (N), acidosis (Ac) and hypoxia (Hx)


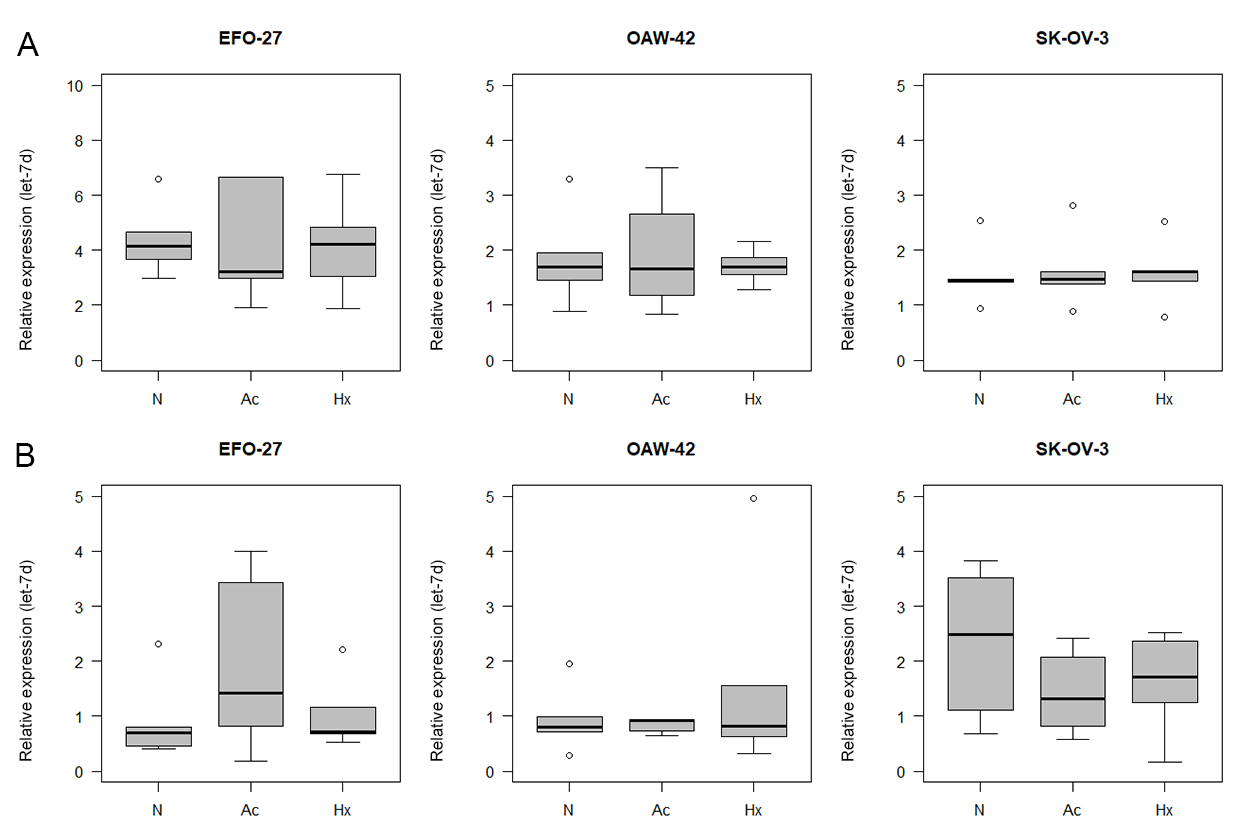


**Supplemental Figure 2:** Expression levels of let-7d intra- (A) and extracellular (B) in the three analyzed cell-lines EFO-27, OAW-42 and SK-OV-3 under normal conditions (N), acidosis (Ac) and hypoxia (Hx)


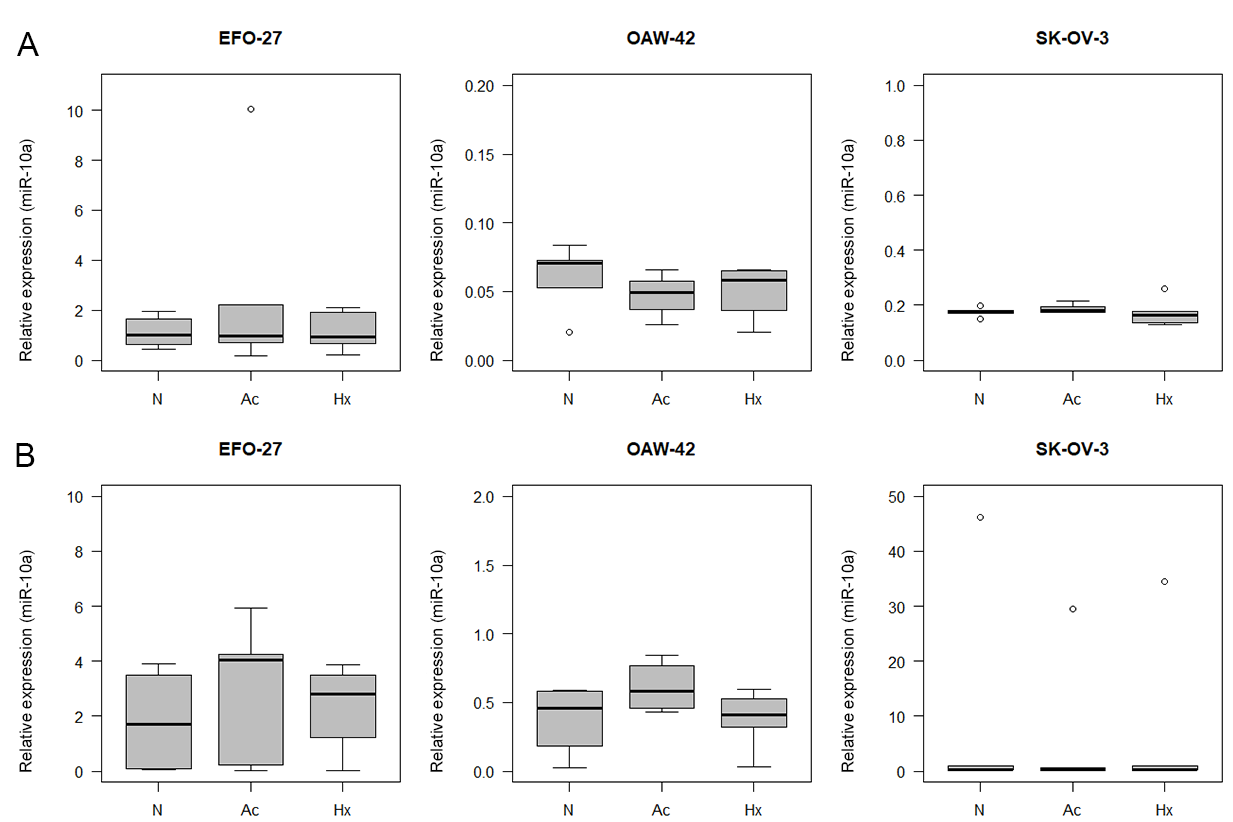


**Supplemental Figure 3:** Expression levels of miR-10a intra- (A) and extracellular (B) in the three analyzed cell-lines EFO-27, OAW-42 and SK-OV-3 under normal conditions (N), acidosis (Ac) and hypoxia (Hx)


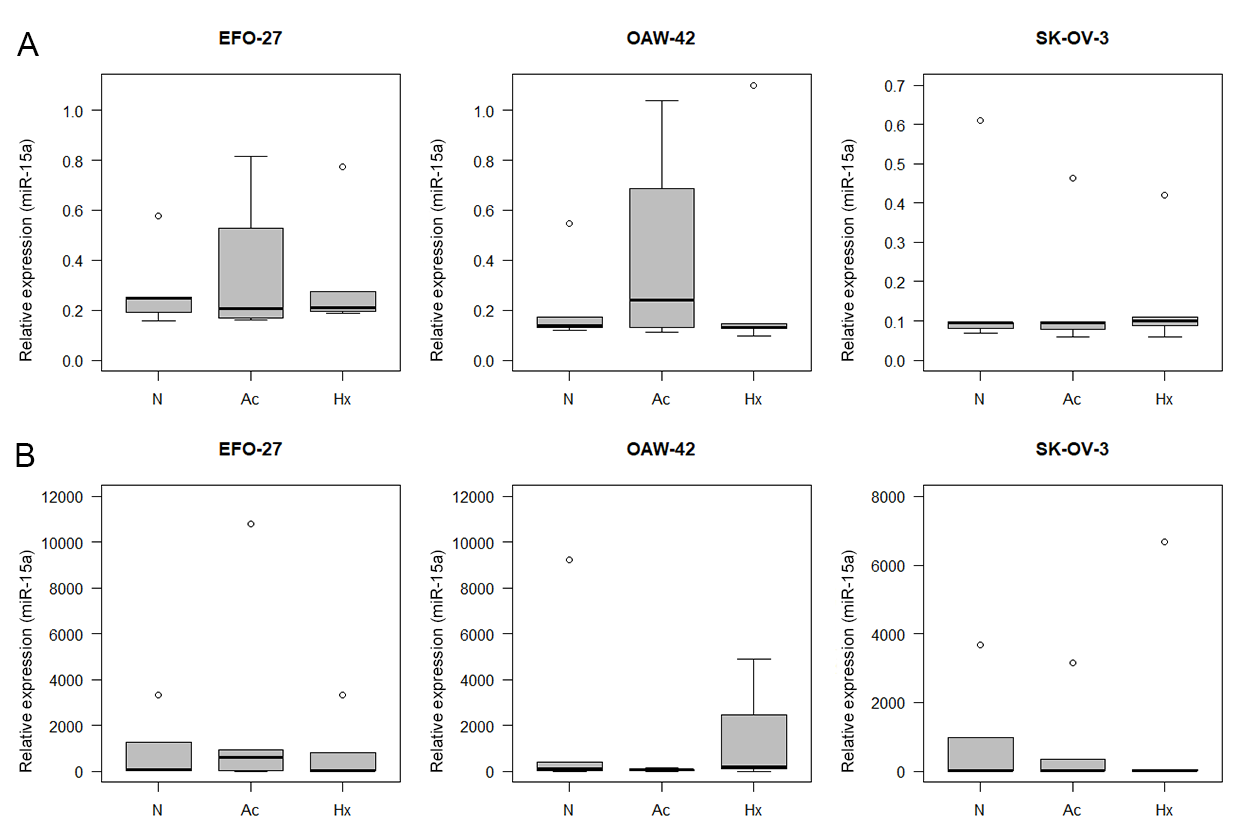


**Supplemental Figure 4:** Expression levels of miR-15a intra- (A) and extracellular (B) in the three analyzed cell-lines EFO-27, OAW-42 and SK-OV-3 under normal conditions (N), acidosis (Ac) and hypoxia (Hx)


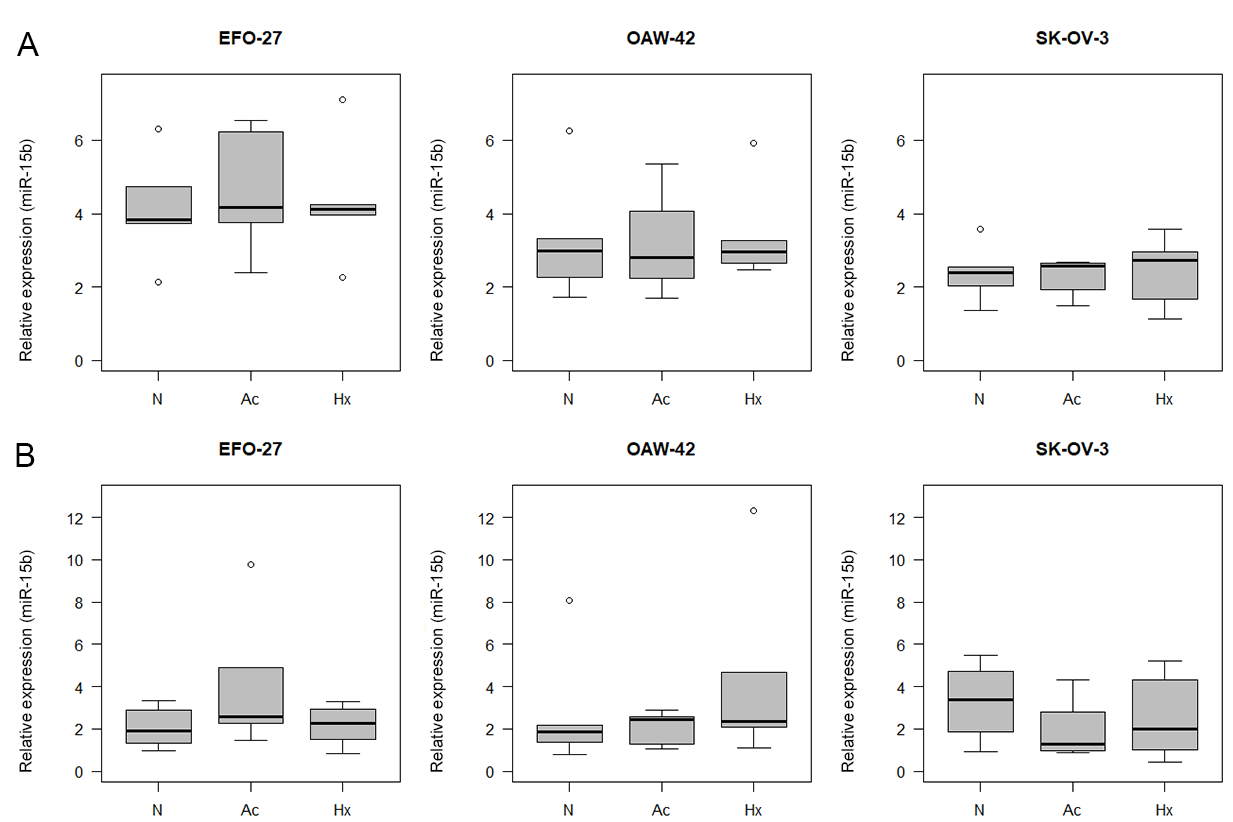


**Supplemental Figure 5:** Expression levels of miR-15b intra- (A) and extracellular (B) in the three analyzed cell-lines EFO-27, OAW-42 and SK-OV-3 under normal conditions (N), acidosis (Ac) and hypoxia (Hx)


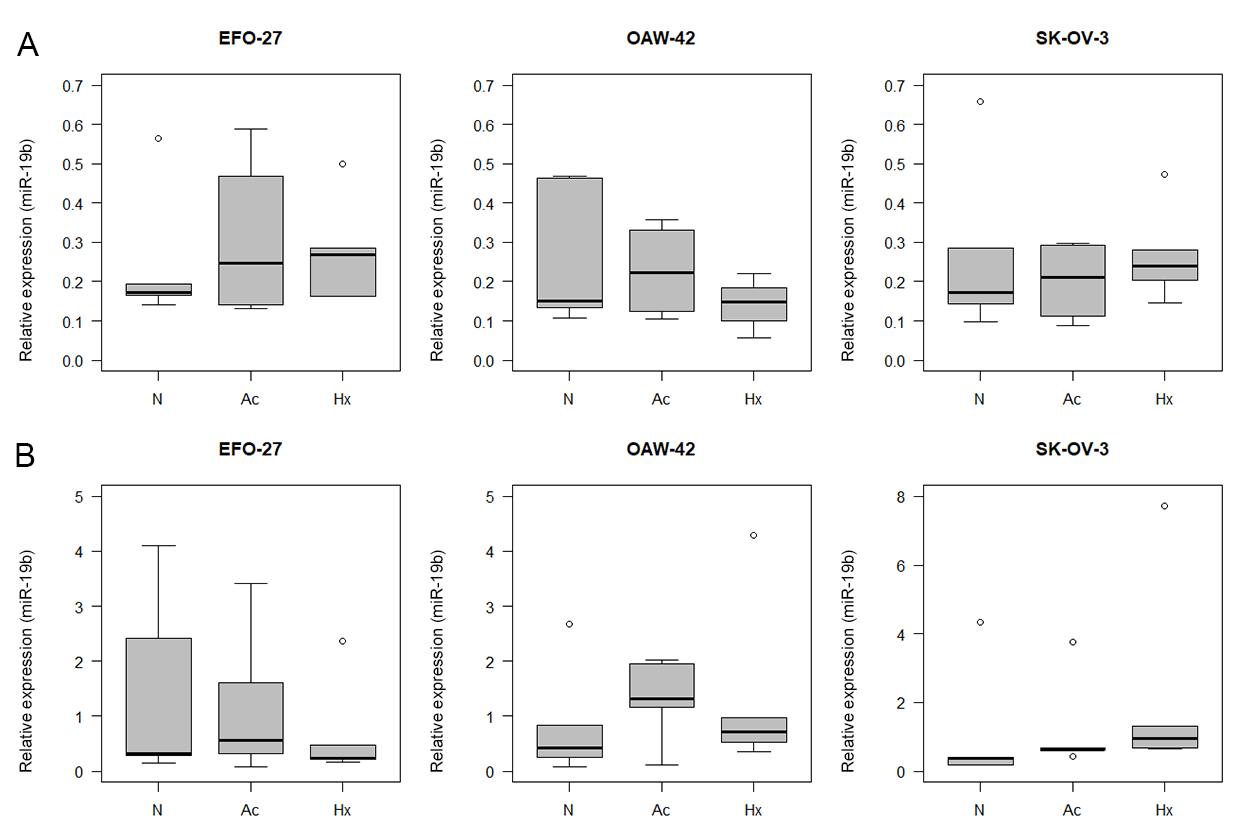


**Supplemental Figure 6:** Expression levels of miR-19b intra- (A) and extracellular (B) in the three analyzed cell-lines EFO-27, OAW-42 and SK-OV-3 under normal conditions (N), acidosis (Ac) and hypoxia (Hx)


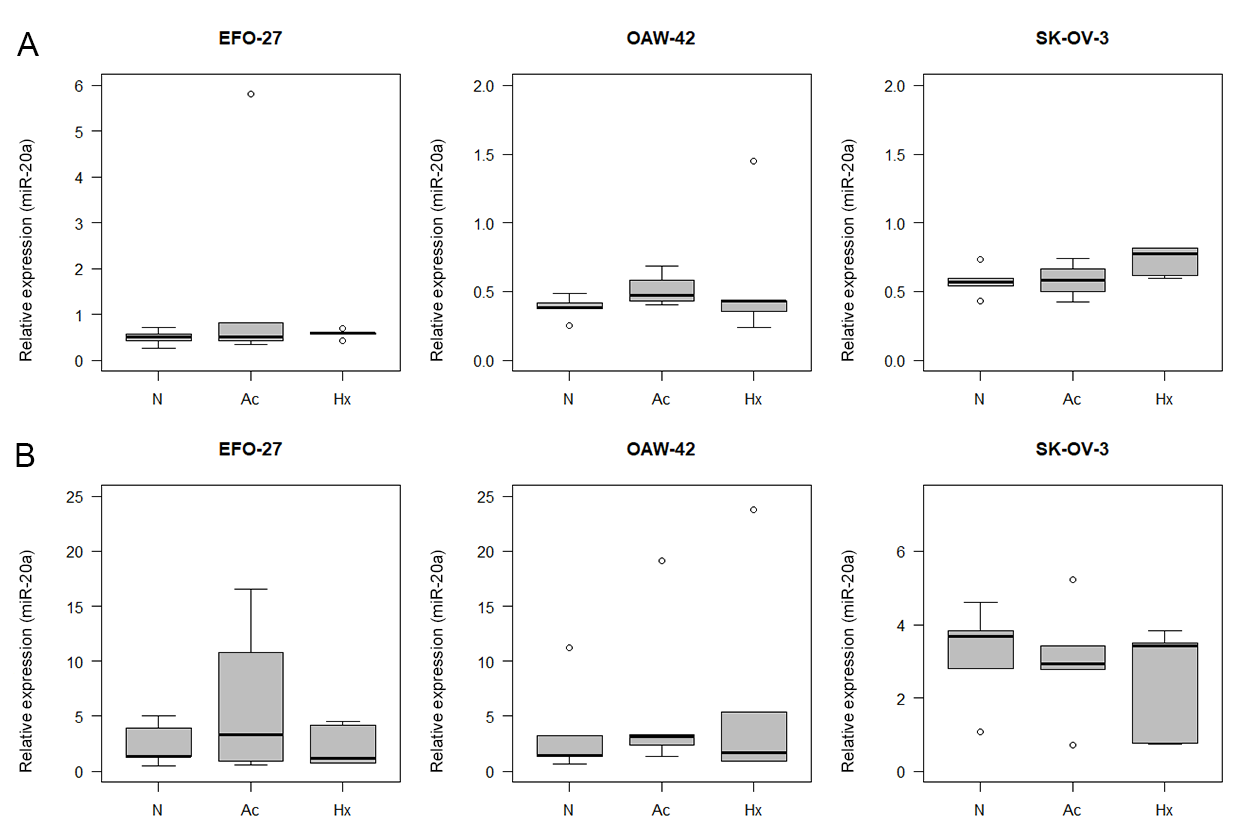


**Supplemental Figure 7:** Expression levels of miR-20a intra- (A) and extracellular (B) in the three analyzed cell-lines EFO-27, OAW-42 and SK-OV-3 under normal conditions (N), acidosis (Ac) and hypoxia (Hx)


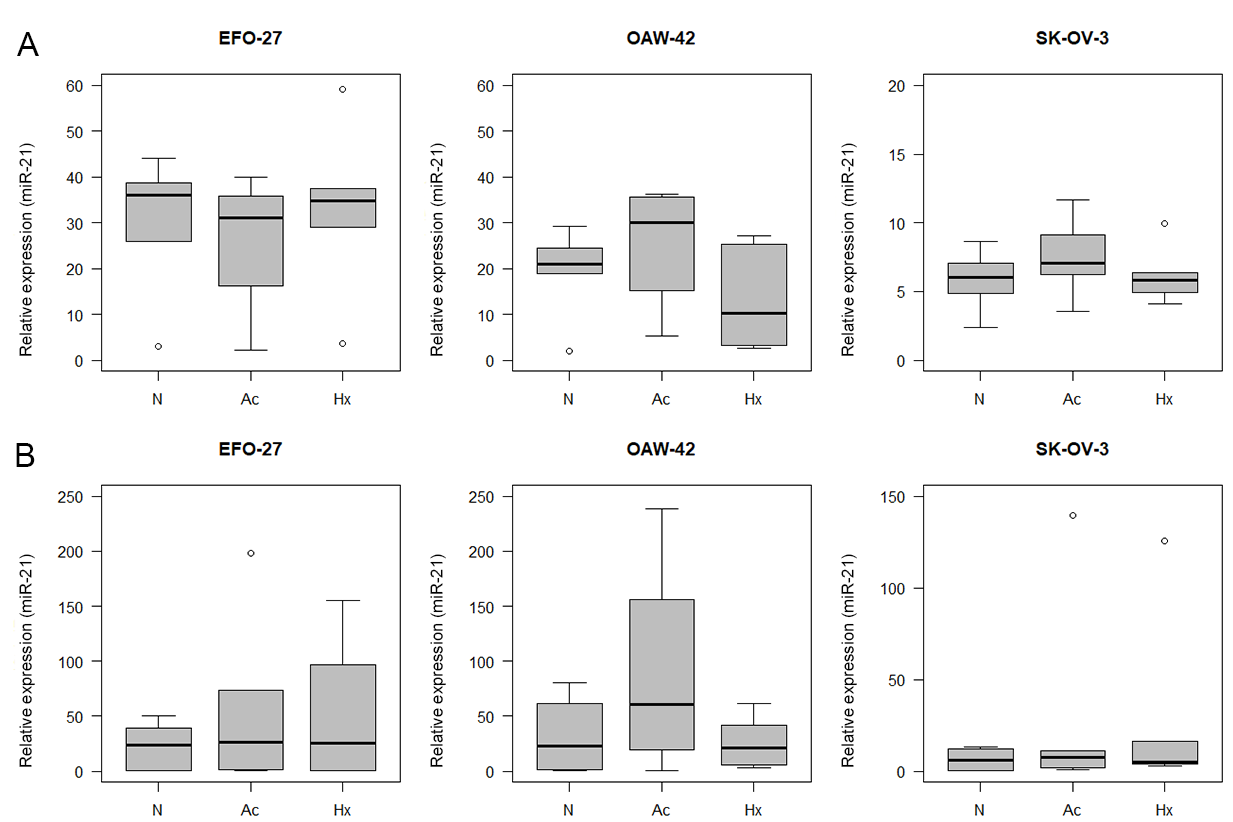


**Supplemental Figure 8:** Expression levels of miR-21 intra- (A) and extracellular (B) in the three analyzed cell-lines EFO-27, OAW-42 and SK-OV-3 under normal conditions (N), acidosis (Ac) and hypoxia (Hx)


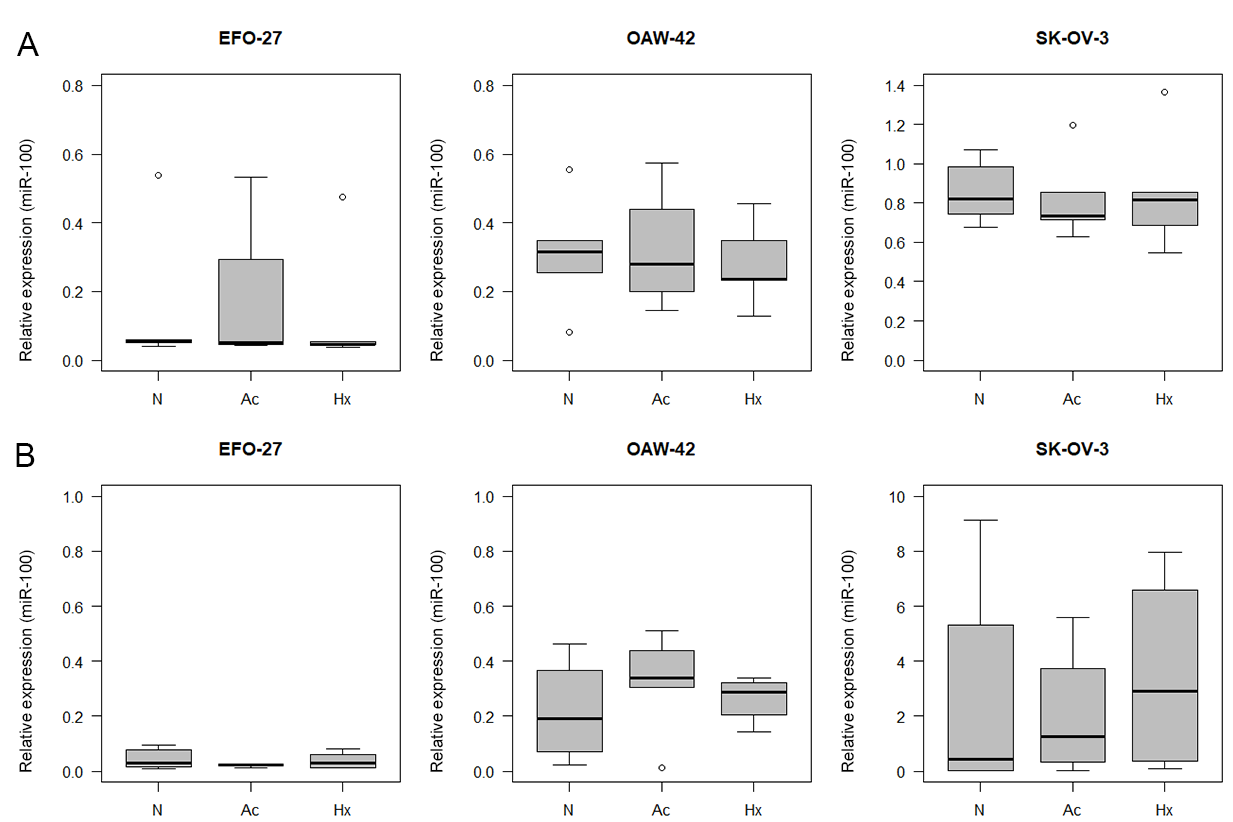


**Supplemental Figure 9:** Expression levels of miR-100 intra- (A) and extracellular (B) in the three analyzed cell-lines EFO-27, OAW-42 and SK-OV-3 under normal conditions (N), acidosis (Ac) and hypoxia (Hx)


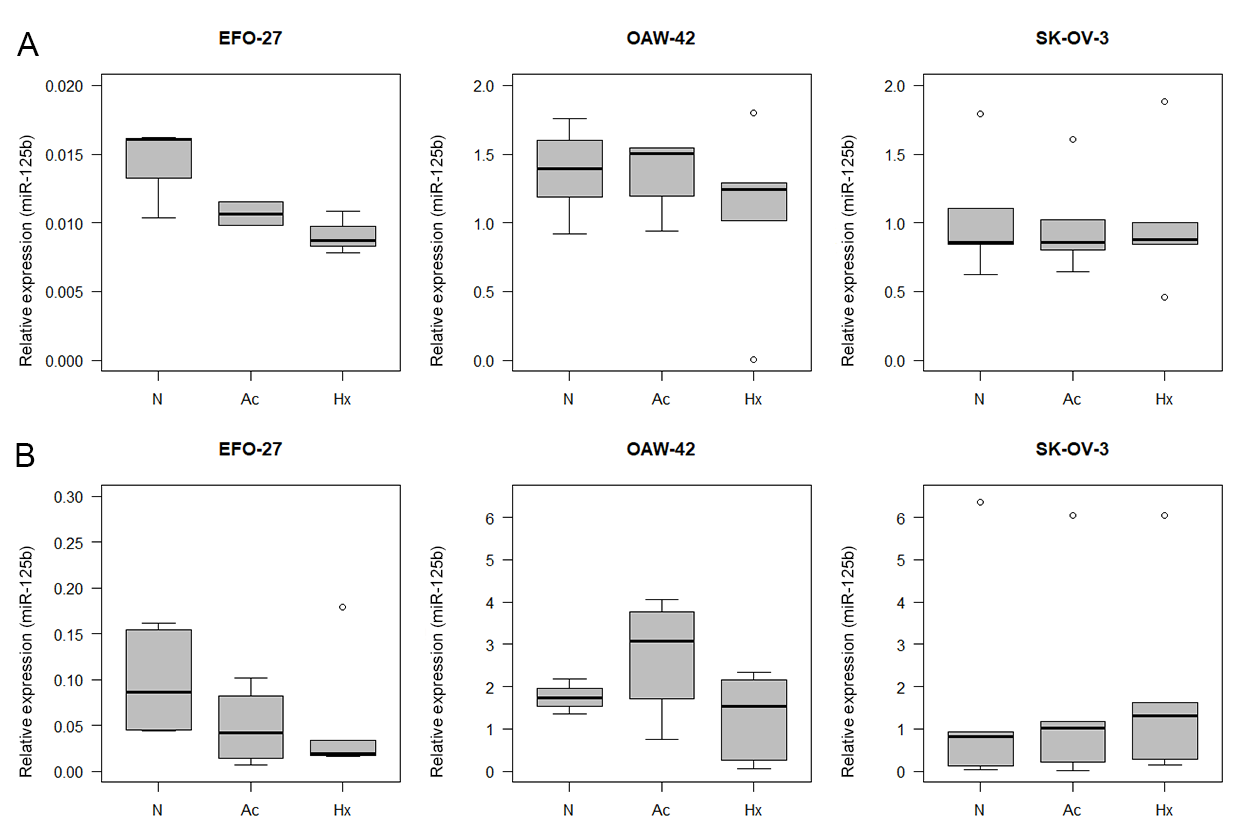


**Supplemental Figure 10:** Expression levels of miR-125b intra- (A) and extracellular (B) in the three analyzed cell-lines EFO-27, OAW-42 and SK-OV-3 under normal conditions (N), acidosis (Ac) and hypoxia (Hx)


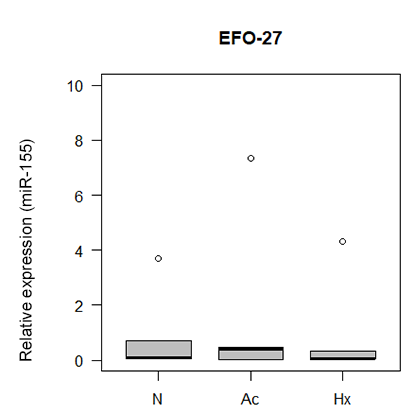

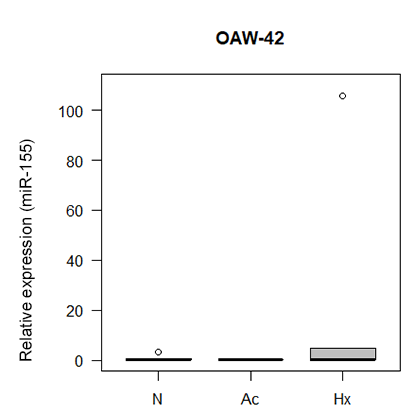

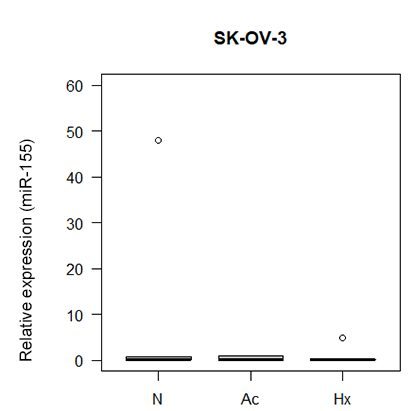


**Supplemental Figure 11:** Expression levels of miR-155 extracellular only in the three analyzed cell-lines EFO-27, OAW-42 and SK-OV-3 under normal conditions (N), acidosis (Ac) and hypoxia (Hx). MiR-155 was not detectable intracellularly.


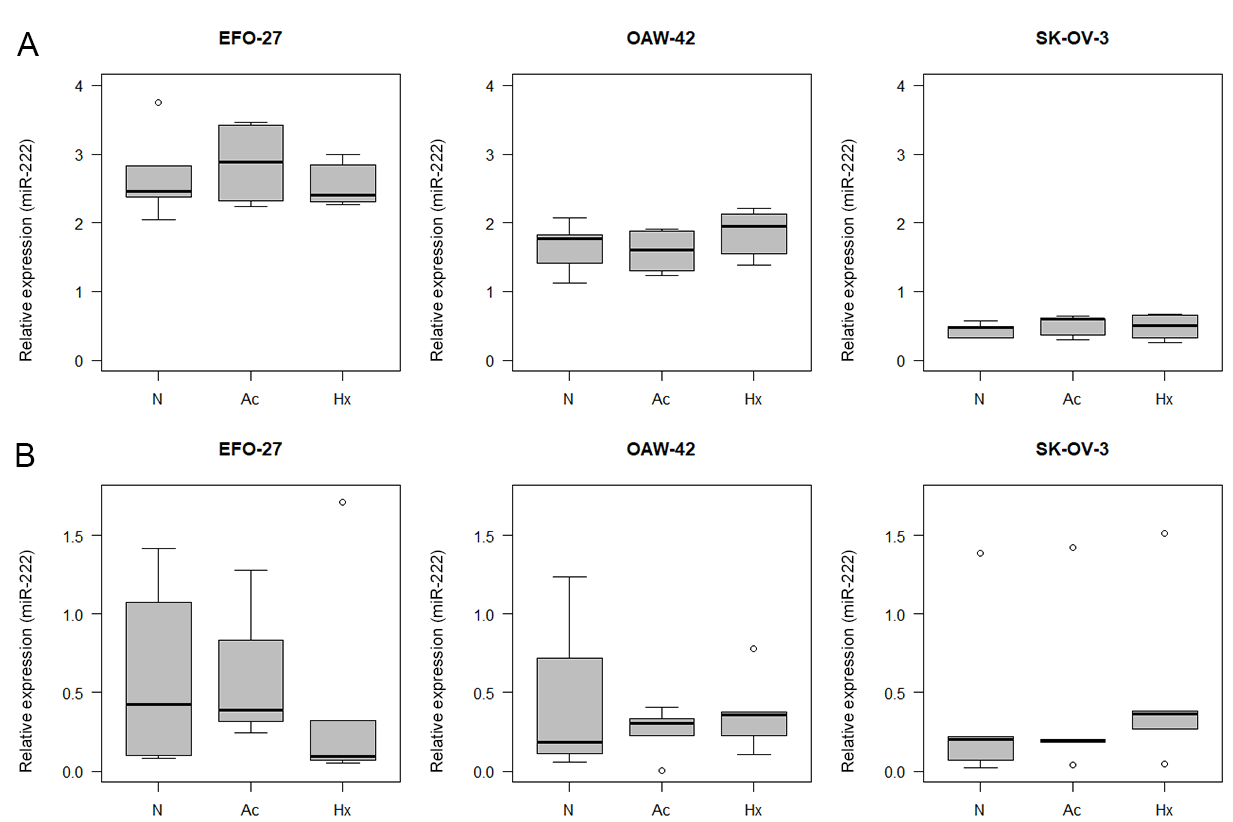


**Supplemental Figure 12:** Expression levels of miR-222 intra- (A) and extracellular (B) in the three analyzed cell-lines EFO-27, OAW-42 and SK-OV-3 under normal conditions (N), acidosis (Ac) and hypoxia (Hx)


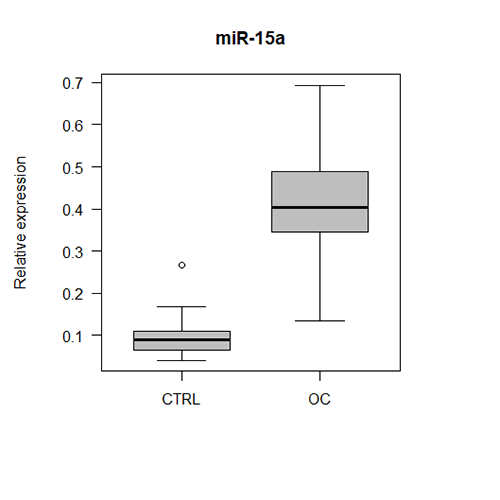

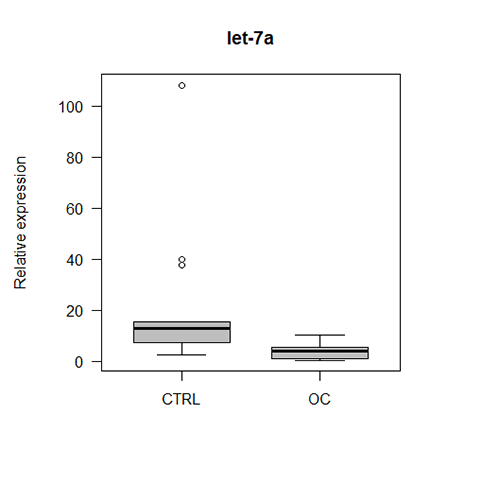

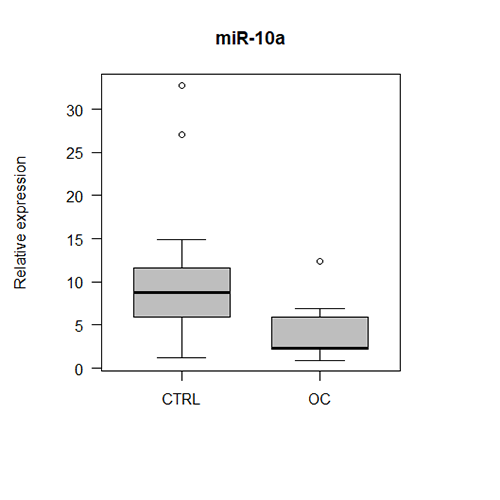


**Supplemental Figure 13:** Significant urinary results shown - Expression levels of miR-15a, let-7a and miR-10a in the urine of OC patients (OC) compared to healthy controls (CTRL)


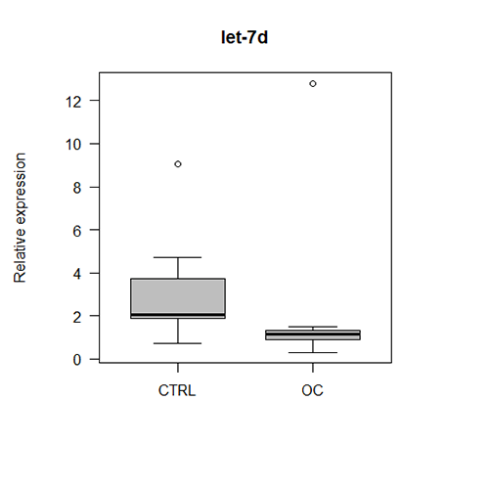

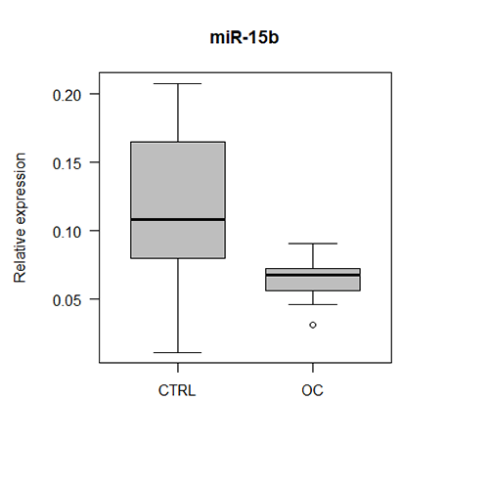

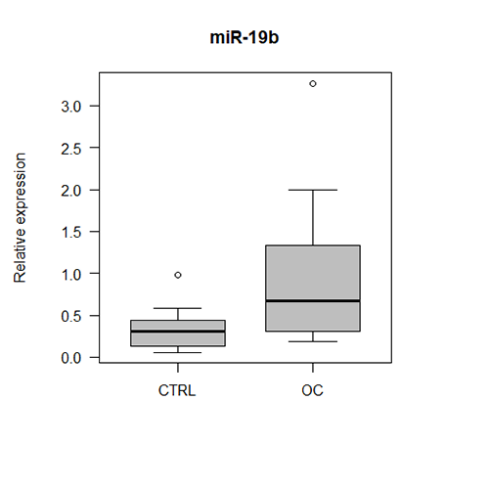

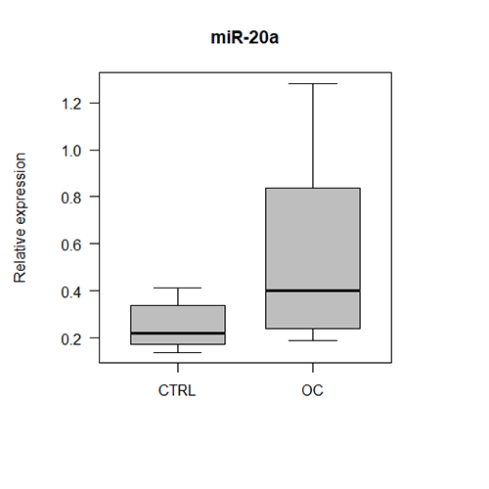

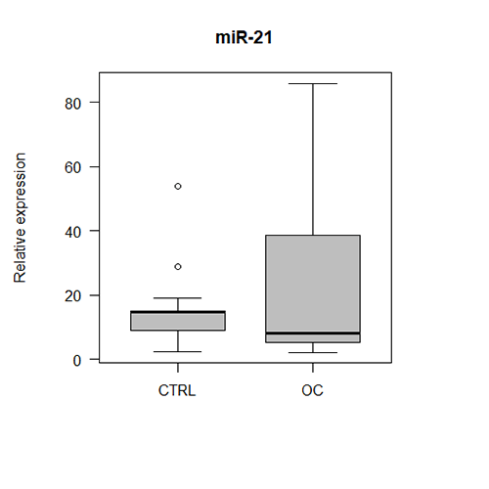

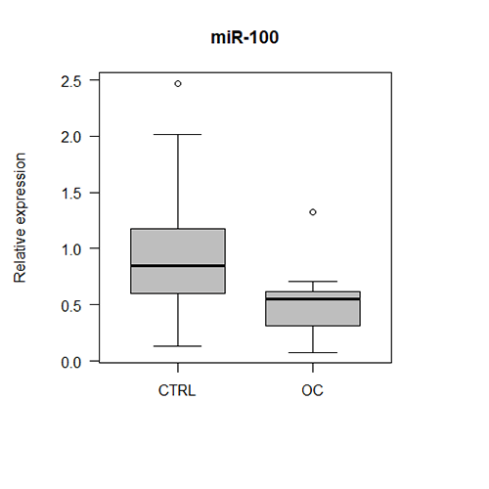

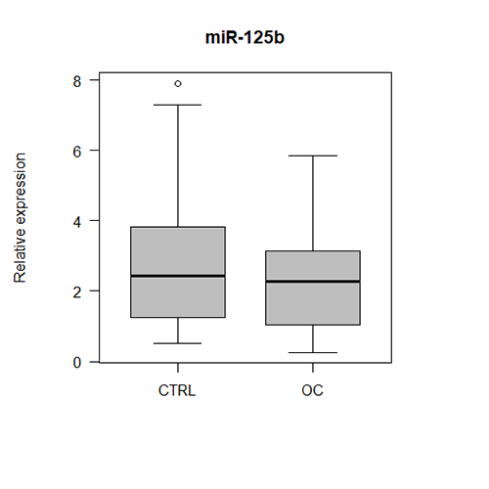

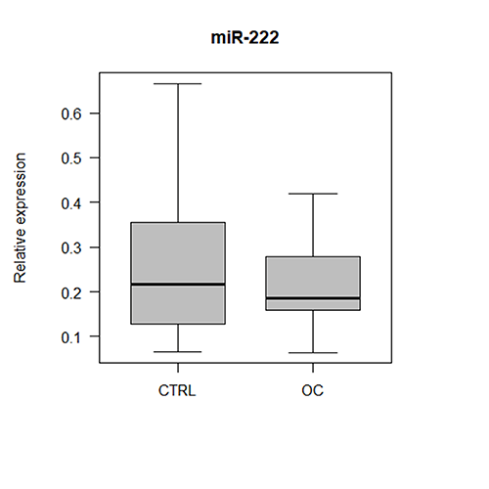


**Supplemental Figure 14:** Non-significant urinary results shown - Expression levels of let-7d, miR-15b, miR-19b, miR-20a, miR-21, miR-100, miR-125b and miR-222 in the urine of OC patients (OC) compared to healthy controls (CTRL). MiR-155 not shown, since it was not detectable in any of the urine samples.
